# 2,4-Dihydr­oxy-*N*′-(3,4,5-trimethoxy­benzyl­idene)benzohydrazide

**DOI:** 10.1107/S1600536809035764

**Published:** 2009-09-09

**Authors:** Liang Xu, Shan-Shan Huang, Bao-Jing Zhang, Shou-Yu Wang, Hou-Li Zhang

**Affiliations:** a College of Pharmacy, Liaoning University of Traditional Chinese Medicine, Dalian 116000, People’s Republic of China; b Dalian Medical University, Dalian 116044, People’s Republic of China

## Abstract

In the title compound, C_17_H_18_N_2_O_6_, the mol­ecule is slightly twisted, with a dihedral angle of 18.1 (2)° between the two benzene rings. In the crystal structure, mol­ecules are linked into a network by inter­molecular N—H⋯O, O—H⋯N and O—H⋯O hydrogen bonds. An intra­molecular O—H⋯O hydrogen bond is also present.

## Related literature

For the biological properties of Schiff base compounds, see: Brückner *et al.* (2000 ▶); Harrop *et al.* (2003 ▶); Ren *et al.* (2002 ▶). For the crystal structures of some Schiff bases and their complexes, see: Diao (2007 ▶); Diao *et al.* (2007 ▶, 2008 ▶); Huang *et al.* (2007 ▶); Li *et al.* (2007 ▶).
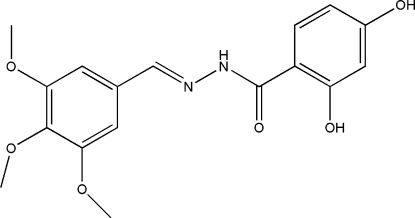


## Experimental

### 

#### Crystal data

C_17_H_18_N_2_O_6_
*M* *_r_* = 346.33
Orthorhombic, 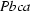

*a* = 14.601 (1) Å
*b* = 11.030 (2) Å
*c* = 20.006 (2) Å
*V* = 3222.0 (7) Å^3^
*Z* = 8
Mo *K*α radiation
μ = 0.11 mm^−1^
*T* = 298 K
0.20 × 0.20 × 0.20 mm

#### Data collection

Bruker SMART CCD area-detector diffractometer
Absorption correction: multi-scan (*SADABS*; Bruker, 2000 ▶) *T*_min_ = 0.978, *T*_max_ = 0.978
18590 measured reflections
3520 independent reflections
2166 reflections with *I* > 2σ(*I*)
*R*_int_ = 0.069

#### Refinement

*R*[*F* ^2^ > 2σ(*F* ^2^)] = 0.048
*wR*(*F* ^2^) = 0.148
*S* = 1.03
3520 reflections
234 parameters
1 restraint
H atoms treated by a mixture of independent and constrained refinement
Δρ_max_ = 0.41 e Å^−3^
Δρ_min_ = −0.24 e Å^−3^

### 

Data collection: *SMART* (Bruker, 2000 ▶); cell refinement: *SAINT* (Bruker, 2000 ▶); data reduction: *SAINT*; program(s) used to solve structure: *SHELXTL* (Sheldrick, 2008 ▶); program(s) used to refine structure: *SHELXTL*; molecular graphics: *SHELXTL*; software used to prepare material for publication: *SHELXTL*.

## Supplementary Material

Crystal structure: contains datablocks global, I. DOI: 10.1107/S1600536809035764/wn2345sup1.cif

Structure factors: contains datablocks I. DOI: 10.1107/S1600536809035764/wn2345Isup2.hkl

Additional supplementary materials:  crystallographic information; 3D view; checkCIF report

## Figures and Tables

**Table 1:** Hydrogen-bond geometry (Å, °)

*D*—H⋯*A*	*D*—H	H⋯*A*	*D*⋯*A*	*D*—H⋯*A*
N1—H1*A*⋯O5^i^	0.893 (10)	2.109 (13)	2.953 (2)	157 (2)
O3—H3⋯N2^ii^	0.82	2.52	3.214 (3)	143
O3—H3⋯O1^ii^	0.82	1.95	2.674 (2)	147
O2—H2⋯O1	0.82	1.79	2.518 (2)	147

Symmetry codes: (i) 
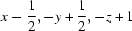
; (ii) 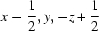.

**Fig. 1.**
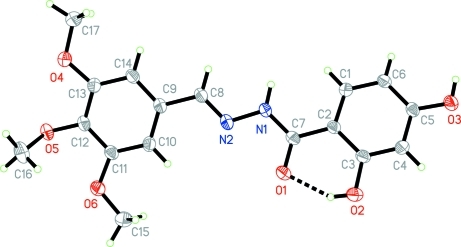
The molecular structure of the title compound, with displacement ellipsoids drawn at the 30% probability level. Hydrogen atoms are represented by spheres of arbitrary radius. The dashed line indicates the intramolecular O—H···O hydrogen bond.

**Fig. 2.**
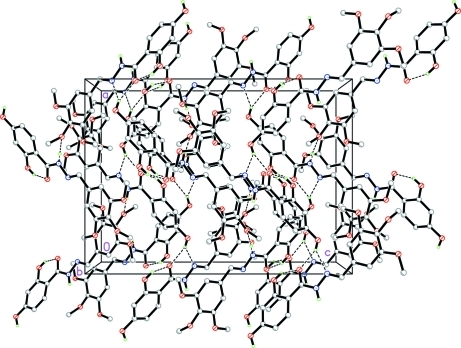
The crystal structure of the title compound, viewed along the b axis. Dashed lines indicate hydrogen bonds. Hydrogen atoms not involved in hydrogen bonding have been omitted.

## supplementary crystallographic information

### Comment

Schiff base compounds have been found to have potential pharmacological and antitumor properties (Brückner *et al.*, 2000; Harrop *et al.*, 2003; Ren *et al.*, 2002). Recently, the crystal structures of a few Schiff base compounds derived from the reaction of aldehydes with benzohydrazides have been reported (Diao *et al.*, 2008; Diao *et al.*, 2007; Diao, 2007; Li *et al.*, 2007; Huang *et al.*, 2007). As a continuation of these studies, we report here the crystal structure of the title compound.

The title compound, C_17_H_18_N_2_O_6_ (Fig. 1) is slightly twisted, with a dihedral angle between the two benzene rings of 18.1 (2)°. The torsion angles C9—C8—N2—N1 and C2—C7—N1—N2 are 2.9 (2) and 7.8 (2)°, respectively. In the crystal structure, molecules are linked into a network (Fig. 2) by intermolecular N—H···O, O—H···N and O—H···O hydrogen bonds (Table 1). An intramolecular O—H···O hydrogen bond is also present.

### Experimental

3,4,5-Trimethoxybenzaldehyde (0.1 mmol, 19.6 mg) and 2,4-dihydroxybenzohydrazide (0.1 mmol, 16.8 mg) were dissolved in a methanol solution (20 ml). The mixture was stirred at reflux for 1 h and cooled to room temperature. After allowing the solution to stand in air for a few days, colorless block-like crystals were formed.

### Refinement

H1A was located in a difference Fourier map and refined isotropically, with the N—H distance restrained to 0.90 (1) Å. Other H atoms were placed in calculated positions and constrained to ride on their parent atoms, with C—H distances of 0.93 and 0.96 Å, an O—H distance of 0.82 Å, and with *U*_iso_(H) = 1.2*U*_eq_(aromatic C) and 1.5*U*_eq_(O and methyl C).

### Crystal data

**Table:**

C_17_H_18_N_2_O_6_	*F*(000) = 1456
*M**_r_* = 346.33	*D*_x_ = 1.428 Mg m^−^^3^
Orthorhombic, *P**b**c**a*	Mo *K*α radiation, λ = 0.71073 Å
Hall symbol: -P 2ac 2ab	Cell parameters from 2734 reflections
*a* = 14.601 (1) Å	θ = 2.5–26.0°
*b* = 11.030 (2) Å	µ = 0.11 mm^−^^1^
*c* = 20.006 (2) Å	*T* = 298 K
*V* = 3222.0 (7) Å^3^	Block, colorless
*Z* = 8	0.20 × 0.20 × 0.20 mm

### Data collection

**Table:**

Bruker SMART CCD area-detector diffractometer	3520 independent reflections
Radiation source: fine-focus sealed tube	2166 reflections with *I* > 2σ(*I*)
graphite	*R*_int_ = 0.069
ω scans	θ_max_ = 27.0°, θ_min_ = 2.0°
Absorption correction: multi-scan (*SADABS*; Bruker, 2000)	*h* = −18→18
*T*_min_ = 0.978, *T*_max_ = 0.978	*k* = −12→14
18590 measured reflections	*l* = −20→25

### Refinement

**Table:**

Refinement on *F*^2^	Primary atom site location: structure-invariant direct methods
Least-squares matrix: full	Secondary atom site location: difference Fourier map
*R*[*F*^2^ > 2σ(*F*^2^)] = 0.048	Hydrogen site location: inferred from neighbouring sites
*wR*(*F*^2^) = 0.148	H atoms treated by a mixture of independent and constrained refinement
*S* = 1.03	*w* = 1/[σ^2^(*F*_o_^2^) + (0.0632*P*)^2^ + 0.9796*P*] where *P* = (*F*_o_^2^ + 2*F*_c_^2^)/3
3520 reflections	(Δ/σ)_max_ = 0.001
234 parameters	Δρ_max_ = 0.41 e Å^−^^3^
1 restraint	Δρ_min_ = −0.24 e Å^−^^3^

### Special details

**Table:**

Geometry. All e.s.d.'s (except the e.s.d. in the dihedral angle between two l.s. planes) are estimated using the full covariance matrix. The cell e.s.d.'s are taken into account individually in the estimation of e.s.d.'s in distances, angles and torsion angles; correlations between e.s.d.'s in cell parameters are only used when they are defined by crystal symmetry. An approximate (isotropic) treatment of cell e.s.d.'s is used for estimating e.s.d.'s involving l.s. planes.
Refinement. Refinement of *F*^2^ against ALL reflections. The weighted *R*-factor *wR* and goodness of fit *S* are based on *F*^2^, conventional *R*-factors *R* are based on *F*, with *F* set to zero for negative *F*^2^. The threshold expression of *F*^2^ > σ(*F*^2^) is used only for calculating *R*-factors(gt) *etc*. and is not relevant to the choice of reflections for refinement. *R*-factors based on *F*^2^ are statistically about twice as large as those based on *F*, and *R*- factors based on ALL data will be even larger.

### Fractional atomic coordinates and isotropic or equivalent isotropic displacement parameters (Å^2^)

**Table:**

	*x*	*y*	*z*	*U*_iso_*/*U*_eq_	
N1	−0.07006 (12)	0.46657 (17)	0.36310 (9)	0.0409 (5)	
N2	0.00500 (12)	0.44220 (16)	0.40282 (9)	0.0410 (4)	
O1	0.01502 (10)	0.60011 (16)	0.30628 (8)	0.0528 (5)	
O2	−0.04899 (12)	0.72863 (18)	0.21335 (9)	0.0650 (5)	
H2	−0.0112	0.7057	0.2409	0.097*	
O3	−0.33269 (11)	0.63390 (17)	0.12189 (8)	0.0555 (5)	
H3	−0.3814	0.6018	0.1322	0.083*	
O4	0.12167 (11)	0.13843 (16)	0.63808 (9)	0.0584 (5)	
O5	0.28830 (10)	0.21803 (14)	0.60976 (8)	0.0474 (4)	
O6	0.31779 (11)	0.37901 (16)	0.51322 (9)	0.0579 (5)	
C1	−0.21013 (15)	0.4894 (2)	0.25893 (12)	0.0420 (5)	
H1	−0.2169	0.4251	0.2886	0.050*	
C2	−0.13151 (14)	0.56173 (19)	0.26278 (11)	0.0384 (5)	
C3	−0.12280 (15)	0.6558 (2)	0.21550 (11)	0.0431 (5)	
C4	−0.19085 (16)	0.6778 (2)	0.16936 (12)	0.0465 (6)	
H4	−0.1843	0.7408	0.1388	0.056*	
C5	−0.26856 (15)	0.6071 (2)	0.16830 (11)	0.0418 (5)	
C6	−0.27758 (15)	0.5110 (2)	0.21243 (11)	0.0421 (5)	
H6	−0.3290	0.4613	0.2106	0.050*	
C7	−0.05838 (14)	0.54391 (19)	0.31173 (11)	0.0396 (5)	
C8	−0.00724 (15)	0.3683 (2)	0.45107 (11)	0.0423 (5)	
H8	−0.0652	0.3366	0.4593	0.051*	
C9	0.07007 (15)	0.33347 (19)	0.49349 (11)	0.0402 (5)	
C10	0.15711 (15)	0.3793 (2)	0.48163 (11)	0.0435 (5)	
H10	0.1665	0.4351	0.4475	0.052*	
C11	0.22944 (15)	0.3418 (2)	0.52056 (12)	0.0437 (6)	
C12	0.21532 (15)	0.2592 (2)	0.57258 (11)	0.0411 (5)	
C13	0.12835 (16)	0.2157 (2)	0.58524 (11)	0.0428 (5)	
C14	0.05539 (15)	0.2521 (2)	0.54515 (11)	0.0428 (5)	
H14	−0.0031	0.2219	0.5530	0.051*	
C15	0.33483 (18)	0.4696 (3)	0.46434 (14)	0.0652 (8)	
H15A	0.3242	0.4367	0.4206	0.098*	
H15B	0.3973	0.4962	0.4677	0.098*	
H15C	0.2946	0.5371	0.4717	0.098*	
C16	0.3270 (2)	0.3053 (2)	0.65403 (14)	0.0655 (8)	
H16A	0.3195	0.3850	0.6355	0.098*	
H16B	0.3910	0.2887	0.6599	0.098*	
H16C	0.2966	0.3010	0.6965	0.098*	
C17	0.03536 (19)	0.0858 (3)	0.65186 (14)	0.0646 (8)	
H17A	−0.0098	0.1486	0.6560	0.097*	
H17B	0.0387	0.0408	0.6929	0.097*	
H17C	0.0186	0.0323	0.6160	0.097*	
H1A	−0.1231 (10)	0.4289 (19)	0.3709 (11)	0.050*	

### Atomic displacement parameters (Å^2^)

**Table:**

	*U*^11^	*U*^22^	*U*^33^	*U*^12^	*U*^13^	*U*^23^
N1	0.0320 (10)	0.0498 (11)	0.0407 (11)	−0.0008 (8)	−0.0063 (8)	0.0046 (8)
N2	0.0349 (10)	0.0496 (10)	0.0385 (11)	0.0046 (8)	−0.0062 (8)	−0.0037 (9)
O1	0.0353 (9)	0.0687 (11)	0.0544 (11)	−0.0083 (8)	−0.0044 (8)	0.0092 (8)
O2	0.0514 (11)	0.0832 (13)	0.0603 (12)	−0.0237 (10)	−0.0062 (8)	0.0223 (10)
O3	0.0411 (9)	0.0797 (12)	0.0457 (10)	−0.0056 (9)	−0.0069 (8)	0.0135 (9)
O4	0.0475 (10)	0.0716 (11)	0.0561 (11)	−0.0065 (8)	−0.0100 (8)	0.0231 (9)
O5	0.0397 (9)	0.0548 (9)	0.0477 (10)	0.0062 (7)	−0.0115 (7)	−0.0020 (8)
O6	0.0359 (9)	0.0830 (12)	0.0548 (11)	−0.0034 (8)	−0.0009 (8)	0.0142 (9)
C1	0.0383 (12)	0.0432 (12)	0.0445 (13)	−0.0004 (9)	0.0003 (10)	0.0051 (10)
C2	0.0321 (11)	0.0461 (12)	0.0371 (12)	0.0001 (9)	0.0016 (9)	0.0015 (10)
C3	0.0340 (12)	0.0543 (13)	0.0411 (13)	−0.0075 (10)	0.0014 (10)	0.0021 (11)
C4	0.0445 (14)	0.0559 (14)	0.0391 (13)	−0.0046 (11)	0.0008 (10)	0.0104 (11)
C5	0.0347 (12)	0.0586 (14)	0.0320 (12)	0.0022 (10)	−0.0010 (9)	−0.0001 (10)
C6	0.0349 (12)	0.0469 (12)	0.0444 (13)	−0.0044 (10)	−0.0017 (10)	0.0005 (11)
C7	0.0305 (11)	0.0465 (12)	0.0417 (13)	−0.0004 (9)	0.0013 (9)	−0.0037 (10)
C8	0.0350 (12)	0.0497 (13)	0.0423 (13)	−0.0002 (10)	−0.0053 (10)	−0.0020 (11)
C9	0.0368 (12)	0.0463 (12)	0.0374 (13)	0.0027 (9)	−0.0051 (10)	−0.0036 (10)
C10	0.0419 (13)	0.0529 (13)	0.0356 (12)	0.0032 (10)	−0.0025 (10)	0.0037 (10)
C11	0.0326 (12)	0.0558 (14)	0.0427 (13)	0.0010 (10)	0.0004 (10)	−0.0020 (11)
C12	0.0354 (12)	0.0479 (12)	0.0399 (13)	0.0062 (10)	−0.0062 (10)	−0.0028 (10)
C13	0.0437 (13)	0.0464 (12)	0.0382 (13)	0.0023 (10)	−0.0032 (10)	0.0016 (10)
C14	0.0338 (12)	0.0519 (13)	0.0428 (13)	−0.0014 (10)	−0.0033 (10)	−0.0012 (11)
C15	0.0477 (16)	0.0815 (19)	0.0664 (18)	−0.0079 (13)	0.0057 (14)	0.0125 (15)
C16	0.0667 (18)	0.0671 (17)	0.0629 (18)	0.0099 (14)	−0.0232 (15)	−0.0177 (14)
C17	0.0556 (16)	0.0703 (17)	0.0678 (18)	−0.0114 (14)	−0.0058 (14)	0.0237 (14)

### Geometric parameters (Å, °)

**Table:**

N1—C7	1.346 (3)	C4—H4	0.9300
N1—N2	1.380 (2)	C5—C6	1.386 (3)
N1—H1A	0.893 (10)	C6—H6	0.9300
N2—C8	1.276 (3)	C8—C9	1.464 (3)
O1—C7	1.243 (2)	C8—H8	0.9300
O2—C3	1.345 (3)	C9—C14	1.385 (3)
O2—H2	0.8200	C9—C10	1.388 (3)
O3—C5	1.351 (3)	C10—C11	1.376 (3)
O3—H3	0.8200	C10—H10	0.9300
O4—C13	1.362 (3)	C11—C12	1.399 (3)
O4—C17	1.415 (3)	C12—C13	1.381 (3)
O5—C12	1.376 (2)	C13—C14	1.393 (3)
O5—C16	1.425 (3)	C14—H14	0.9300
O6—C11	1.362 (3)	C15—H15A	0.9600
O6—C15	1.420 (3)	C15—H15B	0.9600
C1—C6	1.375 (3)	C15—H15C	0.9600
C1—C2	1.400 (3)	C16—H16A	0.9600
C1—H1	0.9300	C16—H16B	0.9600
C2—C3	1.410 (3)	C16—H16C	0.9600
C2—C7	1.462 (3)	C17—H17A	0.9600
C3—C4	1.378 (3)	C17—H17B	0.9600
C4—C5	1.377 (3)	C17—H17C	0.9600
			
C7—N1—N2	117.54 (17)	C10—C9—C8	120.8 (2)
C7—N1—H1A	122.6 (15)	C11—C10—C9	119.8 (2)
N2—N1—H1A	119.8 (15)	C11—C10—H10	120.1
C8—N2—N1	116.67 (18)	C9—C10—H10	120.1
C3—O2—H2	109.5	O6—C11—C10	125.2 (2)
C5—O3—H3	109.5	O6—C11—C12	114.60 (19)
C13—O4—C17	118.17 (18)	C10—C11—C12	120.2 (2)
C12—O5—C16	114.84 (18)	O5—C12—C13	119.9 (2)
C11—O6—C15	116.89 (19)	O5—C12—C11	120.2 (2)
C6—C1—C2	121.7 (2)	C13—C12—C11	119.9 (2)
C6—C1—H1	119.1	O4—C13—C12	115.19 (19)
C2—C1—H1	119.1	O4—C13—C14	124.9 (2)
C1—C2—C3	117.1 (2)	C12—C13—C14	119.9 (2)
C1—C2—C7	124.0 (2)	C9—C14—C13	119.9 (2)
C3—C2—C7	118.85 (19)	C9—C14—H14	120.1
O2—C3—C4	116.8 (2)	C13—C14—H14	120.1
O2—C3—C2	122.2 (2)	O6—C15—H15A	109.5
C4—C3—C2	121.0 (2)	O6—C15—H15B	109.5
C5—C4—C3	120.3 (2)	H15A—C15—H15B	109.5
C5—C4—H4	119.8	O6—C15—H15C	109.5
C3—C4—H4	119.8	H15A—C15—H15C	109.5
O3—C5—C4	117.2 (2)	H15B—C15—H15C	109.5
O3—C5—C6	122.6 (2)	O5—C16—H16A	109.5
C4—C5—C6	120.1 (2)	O5—C16—H16B	109.5
C1—C6—C5	119.7 (2)	H16A—C16—H16B	109.5
C1—C6—H6	120.2	O5—C16—H16C	109.5
C5—C6—H6	120.2	H16A—C16—H16C	109.5
O1—C7—N1	119.5 (2)	H16B—C16—H16C	109.5
O1—C7—C2	120.3 (2)	O4—C17—H17A	109.5
N1—C7—C2	120.25 (19)	O4—C17—H17B	109.5
N2—C8—C9	119.9 (2)	H17A—C17—H17B	109.5
N2—C8—H8	120.1	O4—C17—H17C	109.5
C9—C8—H8	120.1	H17A—C17—H17C	109.5
C14—C9—C10	120.3 (2)	H17B—C17—H17C	109.5
C14—C9—C8	118.9 (2)		

### Hydrogen-bond geometry (Å, °)

**Table:**

*D*—H···*A*	*D*—H	H···*A*	*D*···*A*	*D*—H···*A*
N1—H1A···O5^i^	0.89 (1)	2.11 (1)	2.953 (2)	157 (2)
O3—H3···N2^ii^	0.82	2.52	3.214 (3)	143
O3—H3···O1^ii^	0.82	1.95	2.674 (2)	147
O2—H2···O1	0.82	1.79	2.518 (2)	147

Symmetry codes: (i) *x*−1/2, −*y*+1/2, −*z*+1; (ii) *x*−1/2, *y*, −*z*+1/2.
